# Addressing the Unmet Need for Treatment in Advanced Hepatocellular Carcinoma With Child-Pugh C Cirrhosis: A Case of a Woman With Hepatocellular Carcinoma and Advanced Liver Disease

**DOI:** 10.7759/cureus.88674

**Published:** 2025-07-24

**Authors:** Erin Chamberlain, Joseph M Cleveland

**Affiliations:** 1 Hematology-Oncology, University of California, Los Angeles Health, San Luis Obispo, USA; 2 Medical Oncology, University of California, Los Angeles Health, San Luis Obispo, USA

**Keywords:** advanced cirrhosis, child-pugh c, child-pugh class c, chronic viral hepatitis, hepatitis, hepatocellular carcinoma (hcc), liver-directed therapy, treatment choices

## Abstract

It has been another active year of advances in our understanding and treatment of liver cancer. Here, we explore major research updates discovered throughout the year of 2024. Despite these significant advances, the treatment of patients with hepatocellular carcinoma (HCC) and Child-Pugh class C cirrhosis remains an unmet need.

## Introduction

While ranking the sixth most common cancer in the USA, hepatocellular carcinoma (HCC) ranks as the most common cancer in many countries globally, including sub-Saharan Africa and Southeast Asia, and is the leading cause of cancer deaths in some global regions [1]. Here, we review major advances in HCC discovered in 2024 regarding known risk factors, early diagnosis, and treatment. Despite these advances, we come to understand in this case report one gaping hole, that is, treatment options for patients with advanced hepatocellular carcinoma and advanced liver disease. Aside from liver transplant for those eligible, there exists limited treatment for patients with Child-Pugh C cirrhosis and HCC. All HCC treatment advances within 2024 included patients with earlier stages of liver disease, establishing the treatment of patients with advanced liver disease and HCC as a major unmet need.

## Case presentation

**Figure 1:**
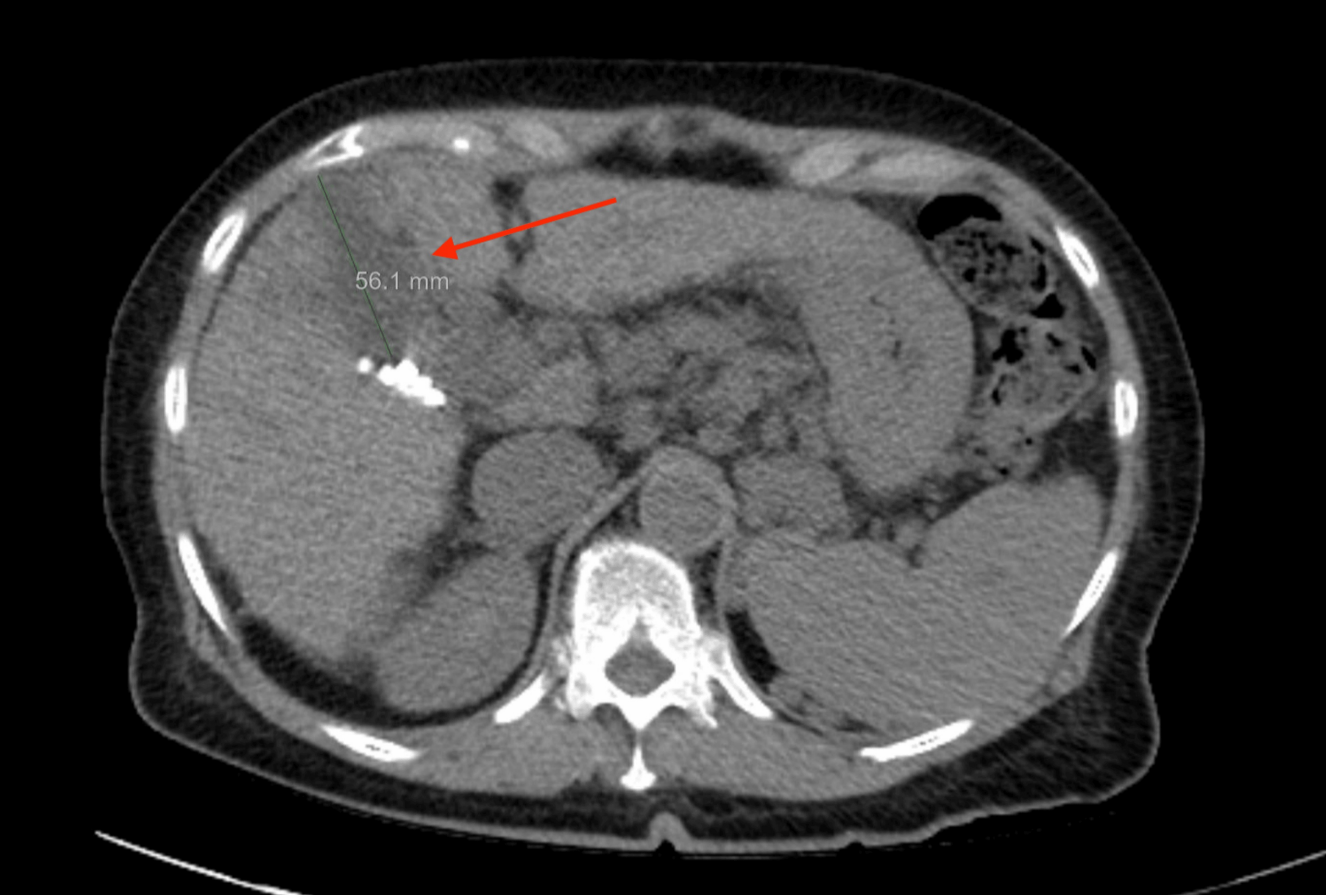
Axial CT of the abdomen and pelvis with contrast revealing suspected HCC (arrow) CT: computed tomography, HCC: hepatocellular carcinoma

**Figure 2:**
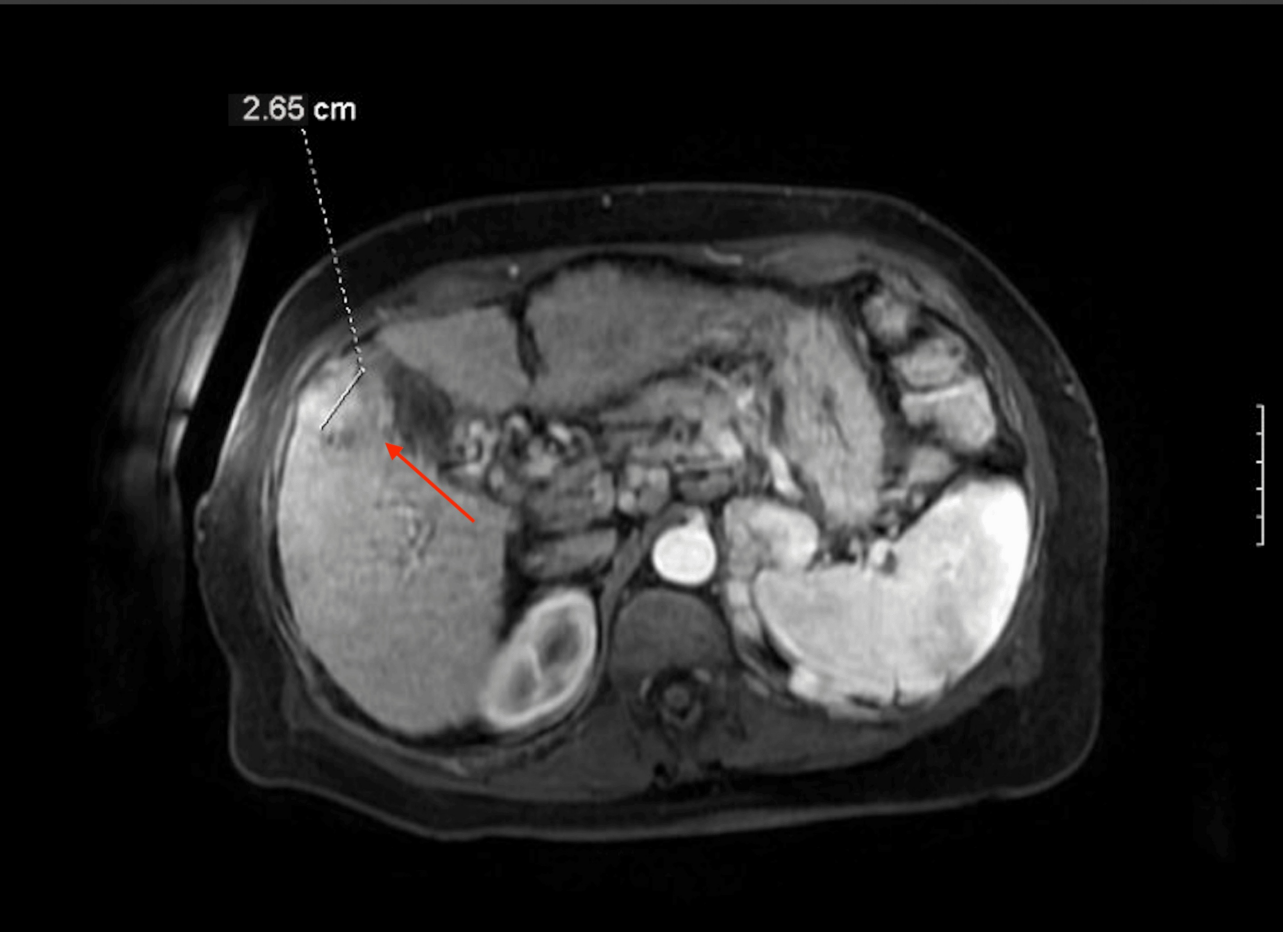
MRI of the abdomen with and without contrast characterizing HCC (arrow) as LI-RADS 5 in segments VIII and V MRI: magnetic resonance imaging, HCC: hepatocellular carcinoma, LI-RADS: Liver Imaging Reporting and Data System

A 72-year-old woman with alcohol dependence, Child-Pugh C cirrhosis, insulin-dependent type 2 diabetes mellitus (DMII), and hypothyroidism presented to the emergency room with coffee ground emesis. She underwent evaluation with laboratory tests, CT imaging, and upper endoscopy and was found to have an acute esophageal variceal bleed. She was stabilized with band ligation and initiation of proton pump inhibitors and beta blockade. Meanwhile, liver function tests demonstrated a total bilirubin of 2.9 mg/dL (normal range: 0.0-1.2 mg/dL), albumin of 3 g/dL (normal range: 3.8-4.8 g/dL), and INR of 1.7 (normal range: 0.9-1.2). Serum alpha-fetoprotein (AFP) measured 171 ng/mL (normal range: 10-40 ng/mL). Additionally, the remaining liver function tests were out of range (Table 1). Furthermore, CT of the abdomen and pelvis with contrast revealed an incidental hepatic mass measuring 5.6 cm and spanning segments VIII and V in the setting of moderate ascites (Figure 1). She subsequently underwent abdominal MRI demonstrating the mass, 2.7 cm in largest dimension, with evidence of portal vein involvement and Liver Imaging Reporting and Data System (LI-RADS) 5 categorization (Figure 2). The smaller tumor size seen on MRI was favored as more accurate, and the discrepancy between imaging modalities was felt to reflect the superior contrast resolution by MRI. No additional regional or distant metastatic disease was seen. She established care with medical oncology and was diagnosed with stage IIIB hepatocellular carcinoma and Child-Pugh C cirrhosis based on elevated bilirubin, low albumin, prolonged INR, ascites, and lack of encephalopathy. Her case was reviewed at a multidisciplinary cancer care conference. Her disease was deemed unresectable, and her case was ineligible for transplant due to macrovascular involvement associated with increased recurrence risk. Furthermore, Child-Pugh C cirrhosis precluded her from liver-directed therapy. She was offered supportive measures with palliative care for symptom management, given no known systemic therapies in the setting of advanced HCC and advanced liver disease.

**Table 1:** Laboratory values during hospitalization AST: aspartate aminotransferase, SGOT: serum glutamic-oxaloacetic transaminase, ALT: alanine aminotransferase, SGPT: serum glutamic pyruvic transaminase, INR: international normalized ratio, AFP: alpha-fetoprotein

Laboratory tests	Patient's results	Normal range	Value interpretation
AST (SGOT)	89 IU/L	0-40 IU/L	Elevated
ALT (SGPT)	44 IU/L	0-32 IU/L	Elevated
Alkaline phosphatase	156 IU/L	44-121 IU/L	Elevated
Bilirubin, total	2.9 mg/dL	0.0-1.2 mg/dL	Elevated
Albumin, serum	3 g/dL	3.8-4.8 g/dL	Decreased
Protein, total, serum	7.9 g/dL	6.0-8.5 g/dL	Normal
INR	1.7	0.9-1.2	Elevated
AFP	171	10-40 ng/mL	Elevated

## Discussion

The patient presented in this case had the risk factor of alcohol dependence and had not been undergoing regular cancer screenings related to paucity of medical care. Established risk factors for hepatocellular carcinoma include viral hepatitis, cirrhosis, obesity, metabolic-associated steatohepatitis, and excessive alcohol consumption, as in this patient's case. In this past year, researchers uncovered an additional risk factor, food insecurity [2]. Screening has been a focus of ongoing research this year, as early treatment of HCC results in 90% five-year survival rates compared to only 20% five-year survival rates associated with treatment of more advanced disease. Eligible individuals for cancer screening include those with hepatitis B viral infection, known Child-Pugh A and B cirrhosis, and Child-Pugh C cirrhosis, and eligible for liver transplant. Current screening methods include hepatic ultrasound imaging combined with serum alpha-fetoprotein (AFP) analysis every six months. This year, investigators developed a novel screening method that combines serum levels of characteristic fusion gene transcription found in HCC (specifically MAN2A1-FER and CCNH-C5orf30 levels) with AFP, producing a 95% accurate early detection of HCC using serum analysis alone. The potential of widespread adoption of this tool for screening holds promise for improving both accurate and early detection of HCC and also replacing the use of more expensive screening imaging procedures [3]. Here, we presented the case of a patient with Child-Pugh C cirrhosis ineligible for transplant. We hope that with the adoption of sensitive and specific screening tools, fewer patients will be faced with a lack of treatment options as in this case.

Extensive progress has been made in treatments for patients with HCC. In 2024 alone, research has resulted in insights and progress in the treatment of patients with HCC. However, an important theme in last year's research is the inclusion criteria of patients with either Child Pugh A and/or B cirrhosis. Patients with Child-Pugh C cirrhosis were excluded on the basis of advanced cirrhosis. Table 2 outlines some of the key studies leading to treatment advances in 2024. Patients with early stages of liver disease will experience improved rates of surgical candidacy, recurrence/relapse-free survival, progression-free survival, overall survival, and quality of life. However, the selection bias in these studies toward patients with early-stage liver disease results in a lack of generalizability to the broader population of all patients diagnosed with HCC (Table 2). Exclusion of patients with advanced liver disease relates to several factors regarding the impact of liver dysfunction on treatment safety and efficacy. The severe liver dysfunction characterized by Child-Pugh C cirrhosis is associated with life-threatening complications such as gastrointestinal bleeding, spontaneous bacterial peritonitis, ascites, and hepatic encephalopathy. Furthermore, it can lead to increased drug toxicity or reduced drug effectiveness due to the compromised ability of the liver to metabolize drugs. Accurate assessment of new treatment effects is challenging to separate from the effects of liver disease progression itself, as Child-Pugh C cirrhosis is a dynamic disease. Lastly, there are ethical concerns related to enrolling this patient population, given the vulnerability of these patients coupled with the potential for serious adverse events and unpredictable outcomes.

**Table 2:** Studies advancing our knowledge regarding treatment of HCC in 2024 HCC: hepatocellular carcinoma, HR: hazard ratio, CI: confidence interval, OS: overall survival, HBV: hepatitis B virus, HCV: hepatitis C virus, TACE: transarterial chemoembolization, PFS: progression-free survival, Y-90: yttrium-90

Study type	Inclusion criteria	Intervention	Outcome	Citation
Retrospective cohort	Child-Pugh A or B cirrhosis and early-stage HCC with high-risk features	Neoadjuvant immune checkpoint inhibitor therapy versus upfront surgical resection	Improved surgical candidacy, conversion rate of 61%, similar recurrence-free survival compared to eligible patients having received upfront surgical resection, 44.8 months versus 49.3 months, p = 0.66, respectively	[4]
Randomized controlled trial	Child-Pugh A or B cirrhosis and high-risk HCC	Adjuvant atezolizumab plus bevacizumab versus active surveillance	Similar median recurrence-free survival when compared to active surveillance alone, 33.2 months versus 36 months, respectively	[5]
Cohort study	Child-Pugh A or B cirrhosis and resectable HCC	Neoadjuvant immune checkpoint inhibitor combination therapy	Patients who achieved either a major or complete pathologic response to neoadjuvant treatment achieved improved median relapse-free survival, not reached at the time of data cutoff versus 28.3 months for patients without a major pathologic response, HR: 0.26, 95% CI: 0.1-0.66, p = 0.0024	[6]
Cohort study	Viral-mediated HCC and no greater than Child-Pugh A cirrhosis	Surgical resection and antiviral therapy	Ten-year OS rates 61% versus 58% for people who did and did not receive HBV antiviral therapy and 82% versus 38% for people who did and did not receive HCV antiviral therapy, both p < 0.001	[7]
Randomized controlled trial	Child-Pugh A or B cirrhosis and embolization-eligible unresectable HCC	TACE alone versus TACE plus durvalumab versus TACE plus durvalumab and bevacizumab	Patients treated with TACE plus durvalumab plus bevacizumab experienced improved PFS versus TACE alone, 15.2 versus 9.2 months, respectively; durvalumab with TACE did not confer improved PFS compared to TACE alone	[8]
Randomized controlled trial	Child-Pugh A cirrhosis	Lenvatinib plus pembrolizumab plus TACE compared to TACE alone	Combined therapy significantly improved PFS, 14.6 months versus 10 months with TACE alone	[9]
Cohort study	Child-Pugh A or B cirrhosis	Varying doses of Y-90 microspheres	Higher doses of Y-90, of at least 150 Gy, improved overall survival by 15 months compared to doses lower than 150 Gy in total; additionally, 17% of patients whose tumor burden was successfully downstaged by Y-90 subsequently underwent conversion therapy with surgical resection, thermal ablation, or liver transplantation and lived four times as long as those who did not receive conversion therapy	[10]
Randomized controlled trial	Child-Pugh A cirrhosis and unresectable HCC	Tremelimumab plus durvalumab versus sorafenib	Tremelimumab plus durvalumab conferred a doubling of five-year survival to 19.6% versus 9.4% with sorafenib; patients treated with tremelimumab plus durvalumab who experienced minor responses of less than 30% tumor shrinkage or stable disease also enjoyed long-term survival benefit	[11]
Randomized controlled trial	Child-Pugh B cirrhosis and unresectable HCC	Tremelimumab plus durvalumab versus sorafenib	Tremelimumab plus durvalumab conferred improved quality of life, functioning, and disease-related symptoms	[12]
Retrospective case series	Child-Pugh B cirrhosis and unresectable HCC	Immune checkpoint inhibitor-based therapy versus best supportive care	Immune checkpoint inhibition plus bevacizumab resulted in improved survival compared to best supportive care, 7.5 months (95% CI: 5.6-11.1 months) versus 4 months ( 95% CI: 3.0-5.0 months), respectively	[13]

Treatments for patients with Child-Pugh C cirrhosis remain an unmet need, and the safety and role of innovative therapeutics remain unknown. With an eye to the future, we can hope that advances in our understanding of the pathophysiology and complications of cirrhosis will lead to the development of more targeted and safer therapies for patients with HCC and advanced liver disease. Furthermore, there is a push to generate more real-world evidence on the effectiveness and safety of therapies in a broader range of patients, rather than highly selected patients that limit relatability, including those with advanced cirrhosis.

## Conclusions

While it has been an amazing year of discovery in the world of hepatocellular carcinoma, during which we gained insights ranging from screening methods, to striking deficits in antiviral therapy definitively linked to HCC survival, to novel approaches to treatment, there remains a paucity of data related to patients with Child-Pugh class C cirrhosis and liver cancer. With modern treatment, patients are enjoying not only improved quality of life but improved survival rates. However, despite these advances, we still lack insight and tools to assist patients living with hepatocellular carcinoma and advanced liver disease. Future trial designs should include patients with severe liver impairment, although this will require careful consideration of potential risks and benefits.
